# An Evaluation and Implementation of Rule-Based Home Energy Management System Using the Rete Algorithm

**DOI:** 10.1155/2014/591478

**Published:** 2014-07-20

**Authors:** Tomoya Kawakami, Naotaka Fujita, Tomoki Yoshihisa, Masahiko Tsukamoto

**Affiliations:** ^1^Graduate School of Engineering, Kobe University, 1-1 Rokkodai-machi, Nada, Kobe, Hyogo 657-8501, Japan; ^2^Cybermedia Center, Osaka University, 5-1 Mihogaoka, Ibaraki, Osaka 567-0047, Japan

## Abstract

In recent years, sensors become popular and Home Energy Management System (HEMS) takes an important role in saving energy without decrease in QoL (Quality of Life). Currently, many rule-based HEMSs have been proposed and almost all of them assume “IF-THEN” rules. The Rete algorithm is a typical pattern matching algorithm for IF-THEN rules. Currently, we have proposed a rule-based Home Energy Management System (HEMS) using the Rete algorithm. In the proposed system, rules for managing energy are processed by smart taps in network, and the loads for processing rules and collecting data are distributed to smart taps. In addition, the number of processes and collecting data are reduced by processing rules based on the Rete algorithm. In this paper, we evaluated the proposed system by simulation. In the simulation environment, rules are processed by a smart tap that relates to the action part of each rule. In addition, we implemented the proposed system as HEMS using smart taps.

## 1. Introduction

In recent years, sensors become popular and various sensors are placed in each home. These homes are called “Smart House,” and many applications such as healthcare are probable [1]. In the Smart House, Home Energy Management System (HEMS) realizes to save energy without decrease in QoL (Quality of Life) by controlling electrical appliances based on those situations [2]. Although the reduction effect of energy consumption depends on the rules, the rule-based HEMS is effective because sensing environment becomes realized and there are many situations to reduce energy consumption of electrical appliances. Thus, HEMS takes an important role in Smart House.

Currently, many rule-based systems to manage energy have been proposed. Almost all of them assume “IF-THEN” rules, and also our research group proposed related method [3, 4]. The IF-THEN rules can be handled in production systems. The Rete algorithm is proposed to speed up the rule processing and is a typical pattern matching algorithm for production systems [5]. Moreover, an approach that distributes the Rete algorithm to some computers has been proposed [6]. Since a large number of electrical appliances, sensors, and rules are expected to exist in each home in the future, this distributed processing model is efficient from the perspective of availability and scalability, for example, building energy management system (BEMS), factory energy management system (FEMS), and community energy management system (CEMS). In addition, the effectiveness of rule-based energy management becomes higher in those large scale and complicated environments. However, the loads for processing rules are probable to concentrate to a specific computer because the existing work aims to distribute a specific part of the processes in the Rete algorithm. Therefore, we have proposed a rule-based HEMS using the Rete algorithm in order to reduce redundant processes and communication loads by the memory of processing results [7]. The proposed system assumes the distributed processing model, and information about electrical appliances and sensors is distributed to “smart taps” [8] in network. The smart tap is a power strip that executes computer programs and controls power feeding for each socket. To manage energy to electrical appliances, smart taps reason situations based on rules. The loads for processing rules and collecting data are distributed to smart taps. In addition, the number of processes and collecting data are reduced by processing rules based on the Rete algorithm. Accordingly, the proposed system enables the rule-based HEMS by low-spec and low-power computing units because the processing loads and the data are distributed.

In this paper, we evaluated the proposed system by simulation. In the simulation environment, rules are processed by a smart tap that relates to the action part of each rule. In addition, we implemented the proposed system as HEMS using smart taps.

In the following, we explain the Rete algorithm and the existing technique in Section 2. The proposed system is described in Section 3. An evaluation of the proposed system is described in Section 4. The implementation of the proposed system is described in Section 5. Finally, we conclude this paper in Section 6.

## 2. The Rete Algorithm

The Rete algorithm is a typical condition matching algorithm for production systems [5]. In the Rete algorithm, a graph structure called Rete network is generated from rules. The Rete network has some types of node to filter data for each condition on rules, such as *α* node and *β* node. The *α* node filters data from single input for each condition on a specific rule. On the other hand, the *β* node joins data from multiple inputs for some matched data on a specific rule. All data are firstly processed on root node of the Rete network, and the data matched conditions of each node are repeated to be processed on the lower nodes. Each node memorizes the matched data and their sets as processing results. By the memory of processing results based on the Rete network, processes for matching condition on rules are reduced also when the data are changed. In addition, the TREAT algorithm has been proposed for an environment where the data are frequently updated [9]. The TREAT algorithm does not have the memories on the Rete algorithm.

**Figure 1:**
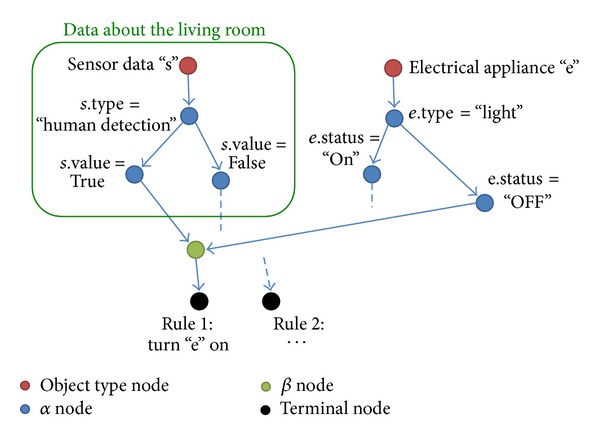
Rete network generated from the rule.

Algorithm 1 shows an example of the rule that represents “turn the lights in the entrance on if someone exists in the living room.” In addition, Figure 1 shows the Rete network generated from the rule of Algorithm 1 on the smart tap placed to the entrance. In the case of Algorithm 1 and Figure 1, data from electrical appliances or sensors are divided to “electrical appliance” or “sensor data” by those object types. After that, the data matched conditions of each node are sent to the lower node. Finally, the data satisfying all conditions on each rule are sent to the node showed as terminal node. The terminal node exists for each rule, and the data matching each rule are retrieved by checking corresponding terminal node. In Figure 1, the data that “someone exists in the living room and the light turned off in the entrance” reaches to the left terminal node. In addition, *α* nodes and *β* nodes memorize the processing results. For example, when the rule which has a condition “no one exists in the living room” is added, new node is added to the lower part of the node shown as “*s*.value = FALSE.” The memory of the existing node is referred and the processes for common condition are reduced.

Currently, an approach that distributes the Rete algorithm to some computers has been proposed [6]. In the existing technique, the load of *α* node on the Rete algorithm is distributed based on Chord [10]. In addition, the related data are shared on publish/subscribe (Pub/Sub) model [11]. However, the existing work aims to distribute the processes of *α* nodes on the Rete algorithm. Thus, the loads for processing rules are probable to concentrate to a specific computer because the processes of *β* nodes and collecting related data such as sensor data are executed by each computer. These redundant processes especially increase communication loads among the computers.

## 3. Proposed System

Currently, we have proposed a rule-based HEMS using the Rete algorithm [7].

### 3.1. Overview

In the proposed system, rules for managing energy are processed by smart taps in network. The loads for processing rules and collecting data are distributed to smart taps. In addition, the number of processes and collecting data are reduced by processing rules based on the Rete algorithm.

**Figure 2:**
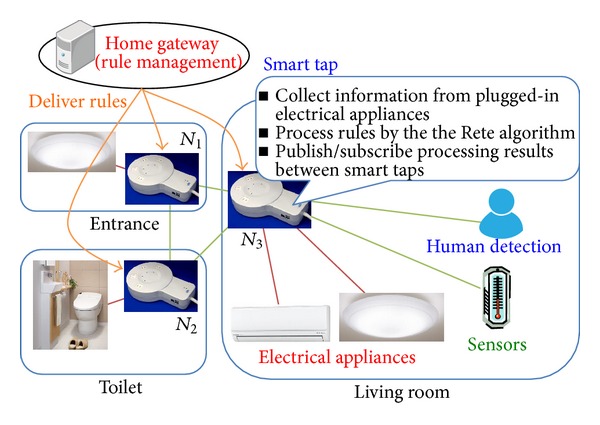
System model.

Figure 2 shows an overview of the proposed system. In the proposed system, smart taps showed as *N*_1_, *N*_2_, and *N*_3_ are put all over the home and connected to LAN to communicate with each other. Electrical appliances are plugged in the smart taps placed near to or in the same room. Each smart tap feeds power to and controls the plugged-in electrical appliances. Sensors are plugged in the smart taps and connected to the LAN or publish observed data via resource networks such as wireless sensor network (WSN) [12–14] and semantic link network (SLN) [15, 16]. The observed data such as user's location [17] are referred from other devices. Smart taps collect related information from electrical appliances and sensors to process IF-THEN rules based on the Rete algorithm. The smart taps memorize the processing results per each condition matching on rules and share the processing results with each other on Pub/Sub model. By sharing the processing results based on the Rete algorithm, the number of processes and collecting data for other rules are reduced. The rules are managed intensively by a specific computer on the LAN. The computer is called home gateway (HG). The HG delivers rules to smart taps when the rules are updated, and all smart taps understand the same rules.

### 3.2. Rule Processing

We describe the flow of rule processing in the case of Algorithm 1 and Figure 1.

In the case of Algorithm 1 and Figure 1, the smart tap *N*_1_ in the entrance processes the rule of Algorithm 1 because action part of the rule is related to electrical appliances in the entrance. Firstly, the smart tap *N*_1_ checks the plugged-in electrical appliances. After that, *N*_1_ collects related data from other smart taps in the living room if *N*_1_ has plugged-in electrical appliances whose “type” is “light” and “status” is “OFF.” In the case of Algorithm 1 and Figure 1, the smart tap *N*_3_ is placed in the living room, and *N*_1_ requests to subscribe related data showed as “*s*.type = TRUE” in Figure 1. *N*_3_ checks the requested data in processing results and publishes matched data to *N*_1_. If *N*_3_ does not have the requested data, *N*_3_ collects the requested data from related sensors and memorizes the processing results. *N*_1_ joins the results of data about “*e*.status = OFF” and “*s*.value = TRUE” from *N*_3_ and turns the lights *e* on if all conditions are satisfied. *N*_1_ memorizes these processing results and keeps them updated with reflecting changes of the data and reprocessing affected parts on rules.

## 4. Evaluation

In this paper, we evaluated the proposed system by simulation.

### 4.1. Simulation Environment

In this simulation, rules are processed by a smart tap that relates to the action part of each rule. As an evaluation value, we measured the number of sending/receiving messages by each node as a load for collecting sensor and processing rules. The load of *N*_*i*_ (*i* = 0,…, *n*) is *L*_*i*_. We used the load of each node to represent total loads of nodes and the maximum load of a node. In addition, the following fairness index (FI) is used as an index for load distribution:

(1) $$\begin{array}{c} \text{FI}=\frac{{\left(\sum_{i=1}^{n}L_{i}\right)}^{2}}{n\sum_{i=1}^{n}L_{i}^{2}}, \end{array}$$

where 0 ≤ FI ≤ 1, and when FI = 1, *L*_0_ = ⋯ = *L*_*n*_. It is indicated that the closer FI is to 1, the more the load is distributed.

Table 1 shows the number of each value in simulation environment. As default values of each parameter, the number of nodes is 2^5^, the number of sensors and rules is 100, and the number of the required sensors per 1 rule is 5. In this simulation, comparison methods to the proposed system “Rete” are “Server” and “Not-Rete.” In the “Server” method, a specific smart tap collects all sensor data and processes rules. In the “Not-Rete” method, each smart tap processes rules and collects related sensor data.

### 4.2. Results by the Number of Sensors

**Figure 3:**
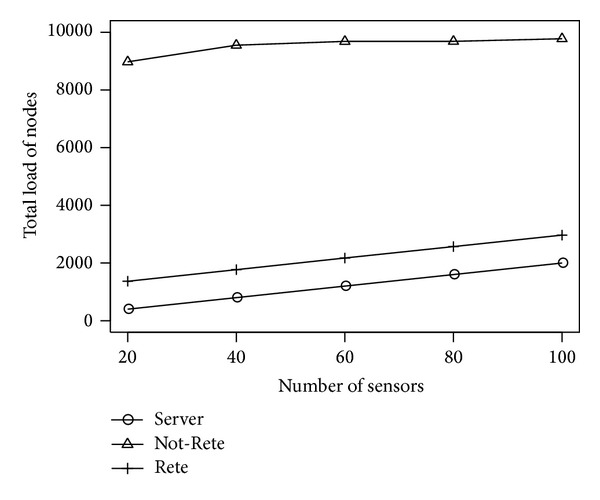
Total load of nodes by the number of sensors.

**Figure 4:**
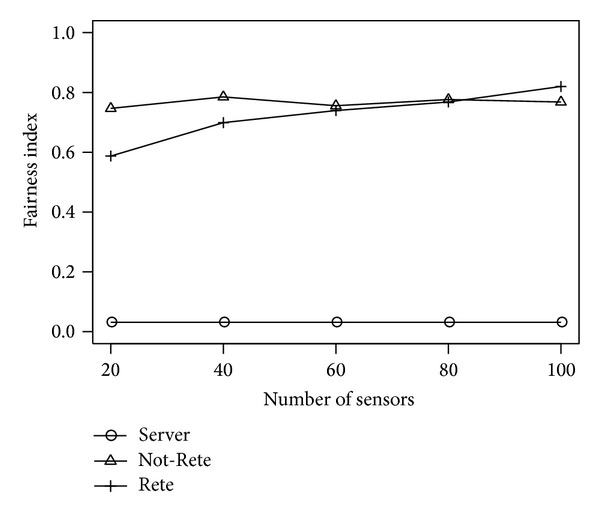
Fairness Index by the number of sensors.

Figure 3 shows the total load of all nodes and Figure 4 shows the fairness index of nodes by the number of sensors. The longitudinal axis is the total load of nodes or fairness index, and the lateral axis is the number of sensors.

In these results, although the number of total sensor data is changed, the amount of sensor data required to process 1 rule is not changed. Therefore, in Not-Rete method, the load of each is always high in this simulation environment that has many rules. On the other hand, in server-based method and Rete-based method in Figure 3, the total load of all nodes is increased by the number of nodes because all sensor data are processed. However, as shown in Figure 4, the Rete-based method distributes the load to nodes widely. Therefore, the average load of node can be controlled by enhancing of nodes based on the system scale.

**Figure 5:**
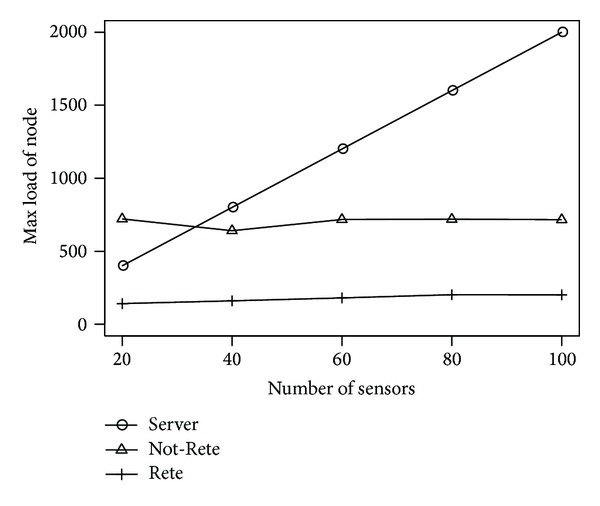
Max load of node by the number of sensors.

Figure 5 shows the maximum load of node. The longitudinal axis is the maximum load of node, and the lateral axis is the number of sensors.

In the Figure 5, the maximum load of node in server-based method is extremely increased by the number of sensors because a specific smart tap collects all sensor data. On the other hand, in Not-Rete method and Rete method, the maximum load of node is not increased largely.

### 4.3. Results by the Number of Rules

**Figure 6:**
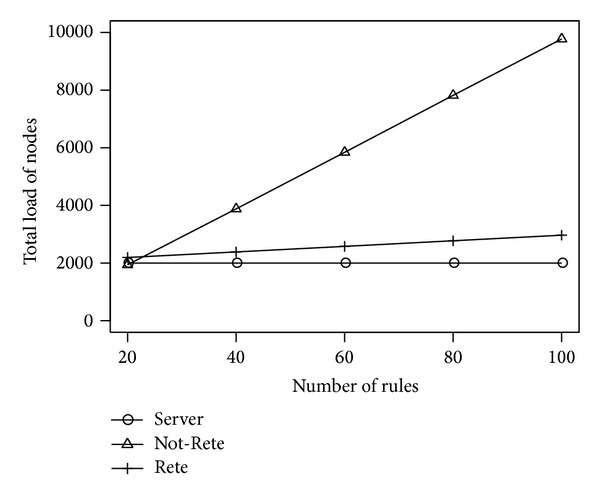
Total load of nodes by the number of rules.

**Figure 7:**
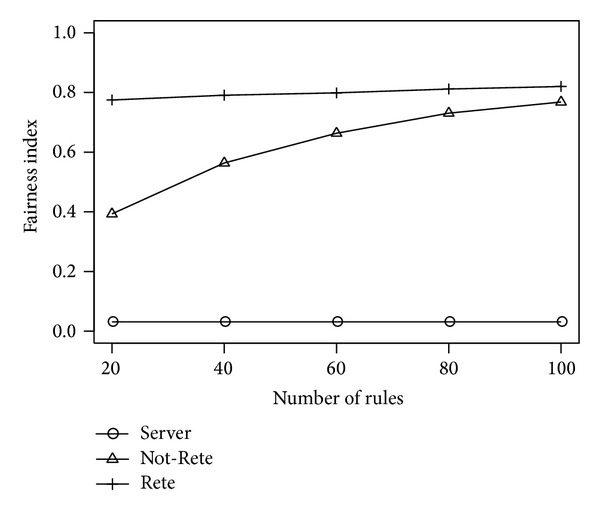
Fairness index by the number of rules.

Figure 6 shows the total load of all nodes and Figure 7 shows the fairness index of nodes by the number of rules. The longitudinal axis is the total load of nodes or fairness index, and the lateral axis is the number of rules.

In Figure 6, the total load of nodes in Not-Rete method is increased because each node processes rules and collects related sensor data individually. On the other hand, in server-based method and Rete-based method, the total load of nodes is not increased largely because the amount of processed sensor data is not increased largely even if the number of rules is increased.

**Figure 8:**
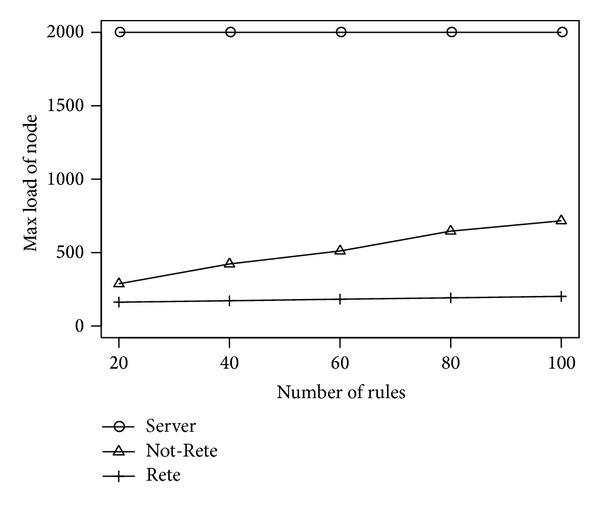
Max load of node by the number of rules.

Figure 8 shows the maximum load of node. The longitudinal axis is the maximum load of node, and the lateral axis is the number of rules.

In Figure 8, the maximum load of node in Not-Rete method is increased because each node processes rules and collects related sensor data individually. On the other hand, in Rete method, the maximum load of node is not increased largely.

### 4.4. Results by the Number of Nodes

**Figure 9:**
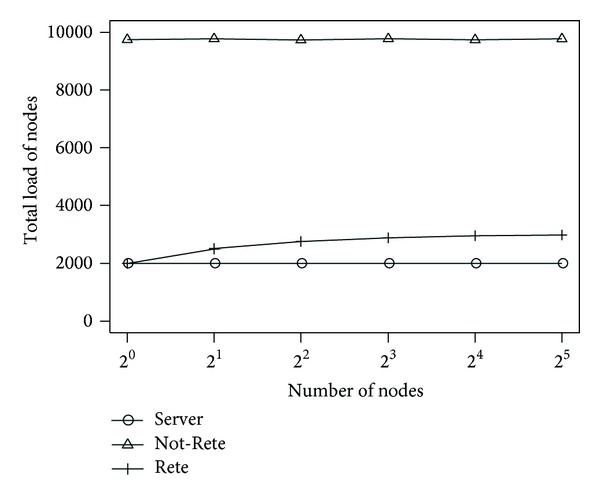
Total load of nodes by the number of nodes.

**Figure 10:**
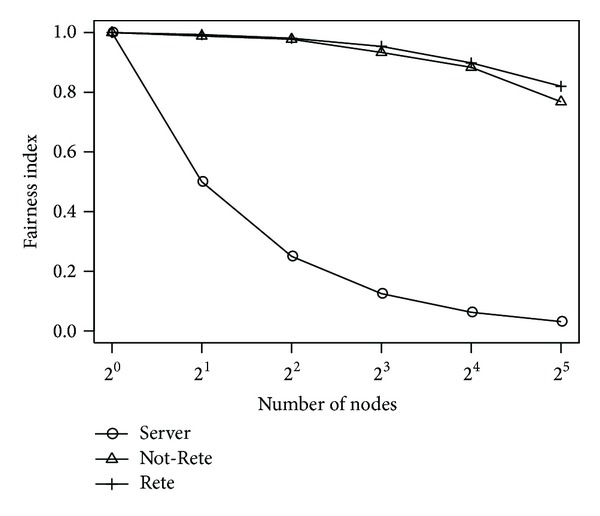
Fairness index by the number of nodes.

Figure 9 shows the total load of all nodes and Figure 10 shows the fairness index of nodes by the number of nodes. The longitudinal axis is the total load of nodes or fairness index, and the lateral axis is the number of nodes.

In these results, the number of total loads of nodes is not changed in server-based method and Not-Rete method. Although the number of total loads is increased in Rete-based method, the difference is not large in this simulation environment. On the other hand, as shown in Figure 10, the fairness indexes of Not-Rete method and Rete-based method are high, and the total load is distributed to the nodes even if the number of nodes is increased.

**Figure 11:**
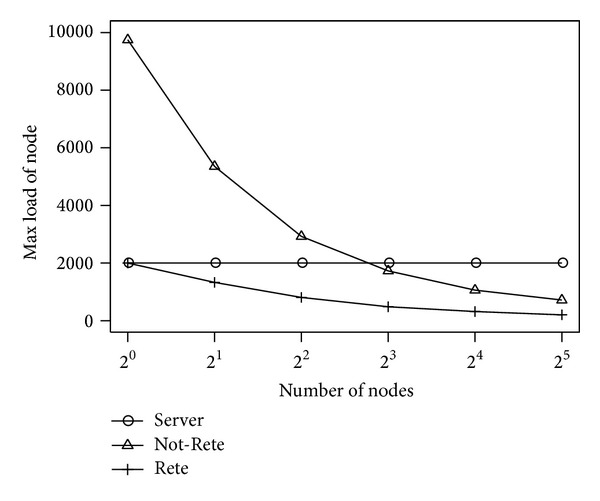
Max load of node by the number of nodes.

Figure 11 shows the maximum load of node. The longitudinal axis is the maximum load of node, and the lateral axis is the number of nodes.

In the Figure 11, the maximum load of node in server-based method is not changed because only one node collects sensor data and processes rules. On the other hand, in Not-Rete method and Rete method, the maximum load of node is decreased by the number of nodes.

### 4.5. Results by the Number of Sensors per 1 Rule

**Figure 12:**
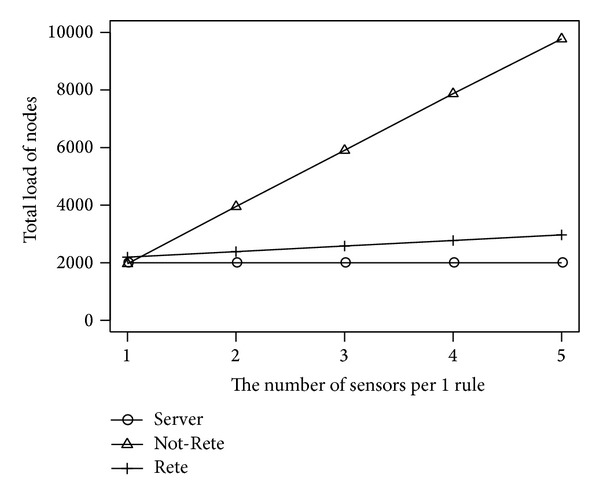
Total load of nodes by the number of sensors per 1 rule.

**Figure 13:**
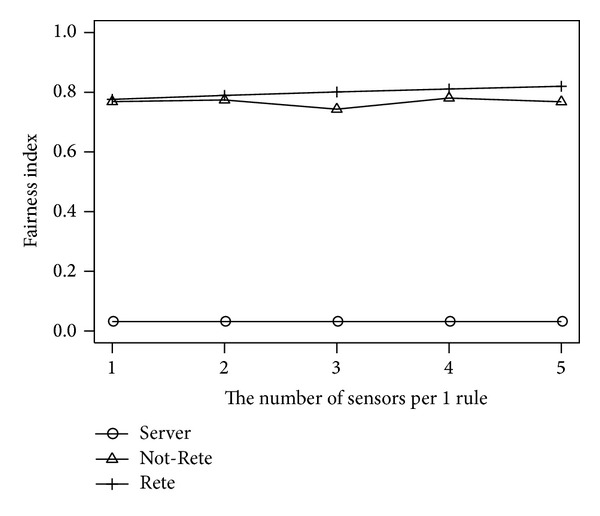
Fairness index by the number of sensors per 1 rule.

Figure 12 shows the total load of all nodes and Figure 13 shows the fairness index of nodes by the number of sensors per 1 rule. The longitudinal axis is the total load of nodes or fairness index, and the lateral axis is the number of sensors per 1 rule.

In Figure 12, similar to Figure 6, the total load of nodes in Not-Rete method is increased because each node processes rules and collects related sensor data individually. On the other hand, in server-based method and Rete-based method, the total load of nodes is not increased largely by the amount of sensor data per 1 rule.

**Figure 14:**
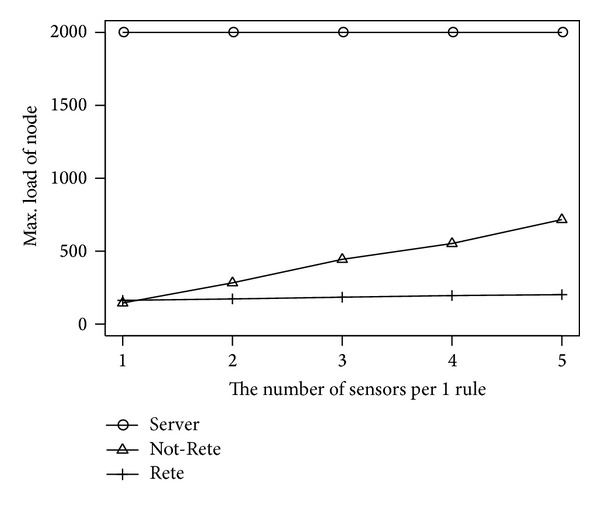
Max load of node by the number of sensors per 1 rule.

Figure 14 shows the maximum load of node. The longitudinal axis is the maximum load of node, and the lateral axis is the number of sensors per 1 rule.

In the Figure 14, similar to Figure 8, the maximum load of node in Not-Rete method is increased because each node processes rules and collects related sensor data individually. On the other hand, in Rete method, the maximum load of node is not increased largely.

## 5. Implementation

In this paper, we describe the implemented system of the proposed system as HEMS.

### 5.1. Smart Tap

We implemented the proposed system as rule-based HEMS using the Rete algorithm and smart tap which has been developed in Enegate Co., Ltd., Japan (Enegate Co., Ltd., available at http://www.enegate.co.jp/ (in Japanese)). The smart tap is developed based on the Raspberry Pi Model-B (Raspberry Pi, available at http://www.raspberrypi.org/). The smart tap has a function of software-based breaker and can switch the power feeding of each socket ON/OFF based on various policies. The smart tap mainly consists of application layer and sensing/control layer.

In the implemented system, the smart taps use data about electric power consumption of plugged-in appliances to manage home energy based on rules. The data are current [A], voltage [V], power [W], and energy [Wh]. The smart tap measures the data per 1 second for each socket.  Algorithm 2 shows an example of the data about electric power consumption described in XML. The function of software-based breaker described above is executed by sending control commands to smart taps also described in XML.  Algorithm 3 shows an example of the command that stops power feeding to socket 1.

### 5.2. Rule Engine

In the proposed system, the decentralized smart taps collect necessary data and process rules autonomously. We have developed the rule engine executed on the smart tap based on Ruleby (Ruleby, available at https://github.com/Ruleby/ruleby/). The Ruleby is described in Ruby and can process the rules based on Rete algorithm. The IF-THEN rule in the Ruleby is also described in Ruby. In the proposed system, we have installed Ruby 1.9.3 and Ruleby to the smart taps.

Table 2 shows the data registered to the rule engine. The data are about plugged-in electrical appliance and sensor data including the data about electric power consumption. In addition, we have defined each data as a class in Ruby.  Algorithm 4 shows an example of the rule described in Ruby. The rule in Algorithm 4 is “if the electric power of TV in the living room is larger than 1 W, the system recognizes that someone exists in the living room and turns on the light.” The proposed system registers the rules such as Algorithm 4 and controls electric appliances based on the rules showed in Algorithm 3.

## 6. Conclusion

In this paper, we evaluated a rule-based Home Energy Management System (HEMS) using the Rete algorithm by simulation. In the simulation results, the proposed system distributes the load for rule processing to nodes and has achieved to reduce the maximum load of node compared with server-based method and Not-Rete method. In addition, we implemented the proposed system as HEMS using smart taps.

In the future, we plan to study the structure of nodes and other assignment methods of rule processing to nodes. For instance, categorization and hierarchization of nodes are probable in more large scale environments. For the other assignment methods, approaches based on frequency of referred rules and performances of node are probable. In addition, we plan to evaluate the proposed system in various environments that have many electrical appliances, rules, and nodes. Eventually, we apply the proposed system to large scale and complicated systems such as BEMS, FEMS, and CEMS.

## Figures and Tables

**Algorithm 1:**
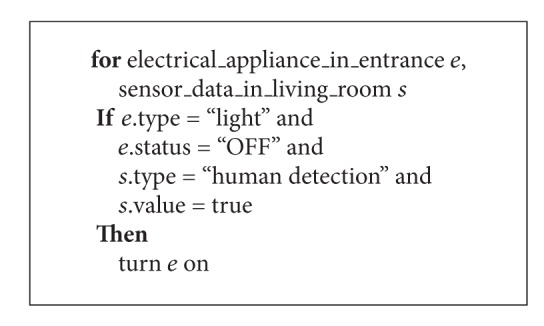
An example of rule.

**Algorithm 2:**
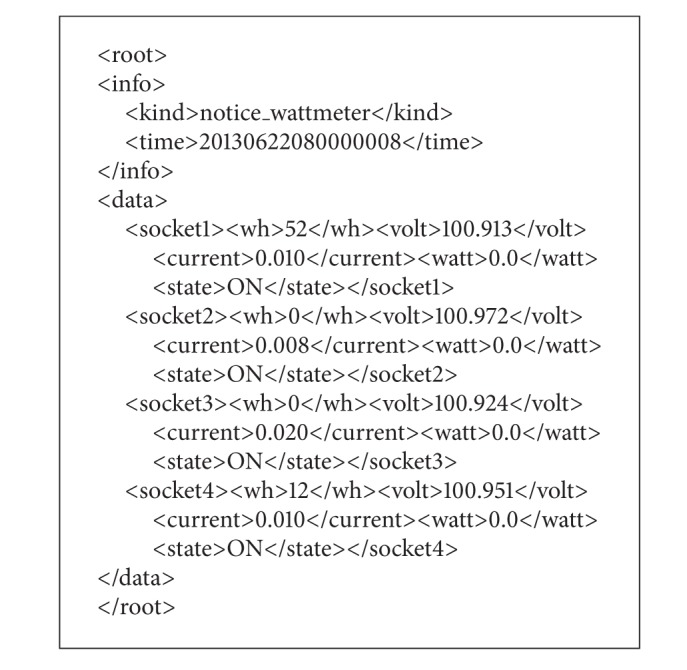
An example of power consumption data.

**Algorithm 3:**
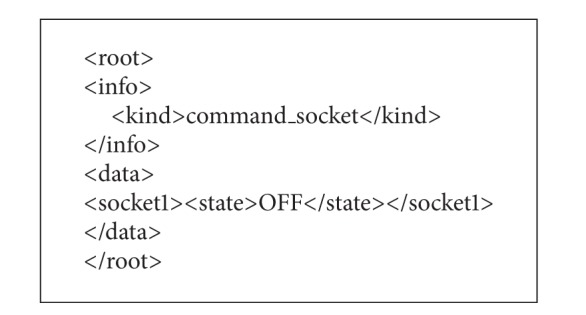
An example of command for the smart tap.

**Algorithm 4:**
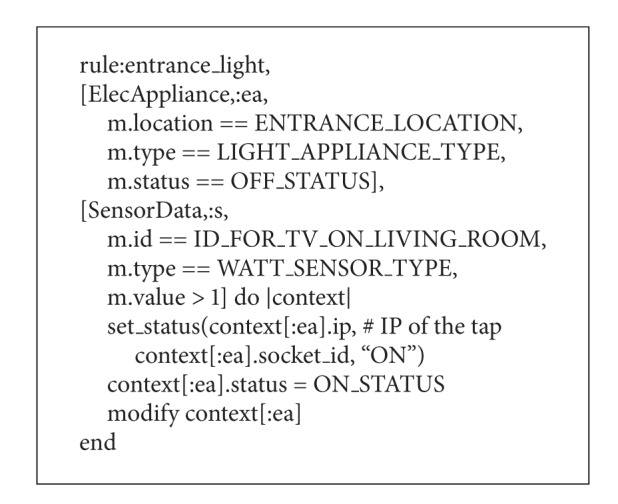
An example of rule in Ruby.

**Table 1:** Simulaiton environment.

Smart taps (nodes)	2^0^, 2^1^, 2^2^, 2^3^, 2^4^, 2^5^
Sensors	20, 40, 60, 80, 100
Sensor data	Sensors × 20
Rules	20, 40, 60, 80, 100
Required sensors per 1 rule	1, 2, 3, 4, 5

**Table 2:** Information registered to rule engine.

Electrical appliances	ID of the plugged-in tap
	IP address of the plugged-in tap
	Socket number (from 1 to 4)
	Type (e.g., TV, light)
	State (e.g., ON, OFF, and ecomode)
	Location
	Priority (e.g., from 1 to 10)

Sensor data	ID
	IP address
	Type (e.g., voltage, temperature)
	State
	Location
	Measured value
	Measurement error
	Measurement time
